# Effect of Seawater Environment on the Structure and Performance of Basalt Continuous Fiber

**DOI:** 10.3390/ma14081862

**Published:** 2021-04-09

**Authors:** Qingwei Wang, Tan Yan, Linfeng Ding

**Affiliations:** 1 State Key Laboratory of Modification of Fiber Materials, Shanghai 201620, China; wqwq888@dhu.edu.cn (Q.W.); 2190330@mail.dhu.edu.cn (T.Y.); 2 Engineering Center of Advanced Glass Manufacturing Technology, Ministry of Education, Donghua University, Shanghai 201620, China

**Keywords:** basalt fiber, seawater environment, chemical durability, tensile strength, microcrack blunting

## Abstract

Basalt continuous fibers (BFs) have been widely applied in the construction industry including marine applications, however, the corrosion mechanism of BFs in a seawater environment is still not well understood. In this work, we explored the effect of the seawater environment on the weight loss, tensile strength, surface morphology, and microstructure structure of BFs via soaking the BFs in seawater solutions at different temperatures and durations. Results show that the weight loss ratio of BFs decreases at the first stage (around 18 h) of soaking at 80 °C, 85 °C, and 90 °C and then increases for longer soaking durations, while the tensile strength has the opposite change. This enhancement of tensile strength and chemical resistance (at the first stage of seawater soaking) is dominated by the ion-exchange induced ‘blunting’ mechanism, even though the results from a Fourier transform infrared (FT-IR) spectrometer and energy-dispersive spectroscopy (EDS) revealed the damaging of the Si–O–Si tetrahedral structure during the corrosion process. This work revealed the full corrosion process and corresponding mechanism of BFs in a seawater environment.

## 1. Introduction

Basalt fiber (BF) is made by continuously drawing from a platinum-rhodium alloy bushing plate [1,2] where the pure basalt rocks are melting at around 1300–1500 °C. Compared with other inorganic fiber materials such as ordinary glass fiber and mineral wool fiber, basalt fiber has good high-temperature resistance and corrosion durability [3,4,5,6], and a similar composition to natural soil, so it has been widely used in reinforced construction materials to improve the mechanical properties and chemical stability of construction [7,8].

A large number of studies [9,10] show that basalt fiber-reinforced polymer reinforcement (BFRP) has good resistance to seawater erosion, and is expected to become an ideal alternative to traditional steel bars. The application of BFRP has the potential to solve the problem of severe corrosion and short service life of marine constructions. Many factors can affect the performance of BFRP in the marine environment, e.g., climate, exposure time, reinforcement method [11], and temperature [12]. Compared with the dry and wet cycle marine environment, the corrosion of BFRP in the immersion environment is much more serious. The increase of ambient temperature will also enhance the degradation and geometric deformation [13] of BFPR rods. Among these, the destruction of the interface between basalt fiber and epoxy resin matrix is the main reason for the degradation of the BFRP sheet [14]. The reaction of Fe^2+^ in basalt fiber with Cl^-^ in seawater, after it is exposed to seawater will accelerate the degradation of BFRP [15]. Moreover, the precipitation of salt will also accelerate the debonding of the fiber–matrix interface, and the hydrolysis of the epoxy resin matrix produces a large number of voids, which accelerates the degradation of the BFRP sheet [16].

Recently, many efforts have been made to improve the durability of BFRP in seawater. Among these, adding nanomaterials, e.g., silanized montmorillonite (Si–MMT) [17], nanotubes (HNTs) [18], etc., to the polymer matrix is one option that can improve the interface interaction between the BF and the polymer matrix, thereby increasing the bonding strength between the enhanced interface [19]. These studies were mostly focused on BFRP composite materials, however, and the performance of BF is also critical especially after the destruction or degradation of BFRP. Few works have examined directly the mechanisms of corrosion of BF in a seawater environment.

In this work, the effect of seawater environment on the surface morphology, structure, and mechanical properties of BFs is explored via immersing basalt fibers in seawater solutions. The mechanism of seawater corrosion on BFs was studied.

## 2. Experiment

### 2.1. Sample Preparation

The basalt fibers (BFs) were prepared by using basalt rocks from Shandong Province (Jiangsu Tianlong Continuous Basalt Fiber Co. Ltd., Yizheng, Jiangsu, China). The basalt rock was placed in a quartz crucible, heated to 1550 °C at a heating rate of ~600 °C/h in a high-temperature furnace, with an isothermal hold for 2 h. Then, the molten basalt melt was rapidly quenched in water to get the basalt glass. The chemical composition of basalt glass (determined by X-ray fluorescence analysis) is shown in Table 1.

Lastly, the continuous BFs were prepared by using a laboratory monofilament fiber drawing system [20] with a platinum crucible at temperatures of ~1350 ± 10 °C and a take-up reel at a rotation speed of ~200 rpm.

### 2.2. Characterization

The seawater solution was prepared based on the ASTM D1141-98 (2013) [21] to simulate the marine environment. The composition is shown in Table 2 to create a seawater corrosion environment for aging experiments.

The accelerated aging method [22] was adopted in the whole process, where the seawater solution was heated to 80–90 °C to accelerate the aging rate of basalt fiber. The whole seawater aging process can be presented as follows: first, the BFs samples were dried at 100 °C for 2 h (h) before weighed and put into three glass beakers (0.1 g each); Then, the seawater solution was poured into the glass beakers to ensure that the BF samples were completely immersed in the seawater solution; then, the glass beakers were sealed with plastic wraps and fixed with rubber bands; finally, the glass beakers were placed into three ovens at temperatures of 80 °C, 85 °C, and 90 °C to start the aging experiment.

The weight loss of the BFs sample was measured after the aging experiment was processed for 6 h, 12 h, 18 h, 24 h, and 168 h (7 days). The BF samples were washed with distilled water 5 times and dried at 100 °C for 2 h before weighing in an analytical balance (Precisa, Zurich, Switzerland, ±0.0001 g). Meanwhile, a 1 mL seawater solution was also tested by inductively coupled plasma spectroscopy (ICP, Prodigy, Hudson, NH, USA).

The tensile strength of basalt fiber was tested by an electronic single fiber dynamometer (YG005A, Wenzhou, China) and 20 samples with diameters of about 22 ± 2 μm were taken from each group to test the tensile strength. A single filament BF sample with a length of 20 ± 1 mm was fixed at the dynamometer and then stretching uniaxially at a speed of 5 mm/min until failure. The tensile strength (*S*, MPa) is calculated based on:

(1) $$S=10000F/\left(\frac{\pi }{4}d^{2}\right)$$

where *F* is the failure strength in cN and d is the diameter of BF in μm.

The morphology and surface composition of the BFs before and after the aging experiment were measured by a field-emission scanning electron microscope (SEM, JSM-7500F, Tokyo, Japan) assisted with energy-dispersive spectroscopy (EDS). The microstructure of BFs after seawater aging was characterized by a Fourier transform infrared spectrometer (FT-IR, Nicolet 8700, Madison, WI, USA).

## 3. Result and Analysis

### 3.1. Weight-Loss Ratio

**Figure 1:**
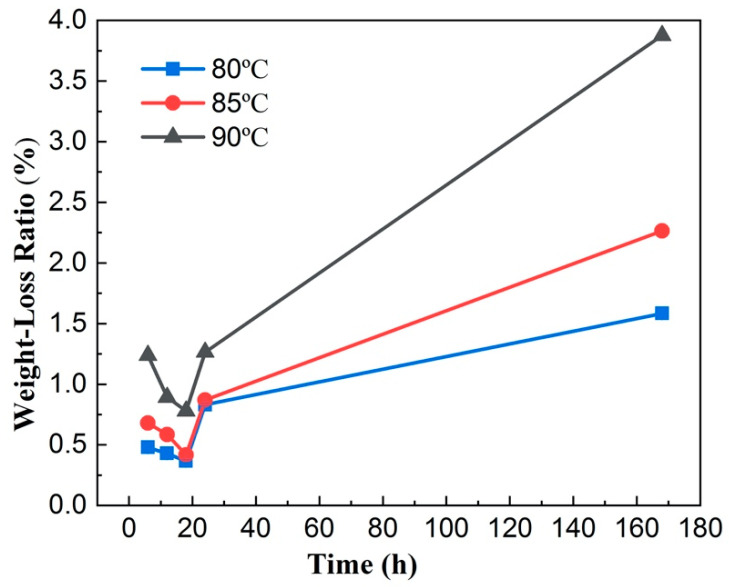
Weight-loss ratio of basalt fiber after soaking in seawater solution for 168 h at 80 °C, 85 °C, and 90 °C. The error bars are within the size of symbols.

The weight-loss ratio (mass loss divided by the initial mass) of BFs after soaking in seawater solution for up to 168 h at 80 °C, 85 °C and 90 °C is shown in Figure 1. In general, the overall weight-loss rate of BFs increases with the increasing temperature of seawater solution from 80 °C to 90 °C and exhibits a similar trend as a function of soaking time. In particular, the mass loss rate of BFs decreases gradually at the first 18 h and starts to increase afterward. For example, the weight-loss rate of the BFs sample after soaking at 85 °C seawater solution for 18 h is only 0.42%, but that of the fiber at the same conditions for 24 h increases to 0.87% and it further increases to 2.26% at 168 h.

### 3.2. Tensile Strength

**Figure 2:**
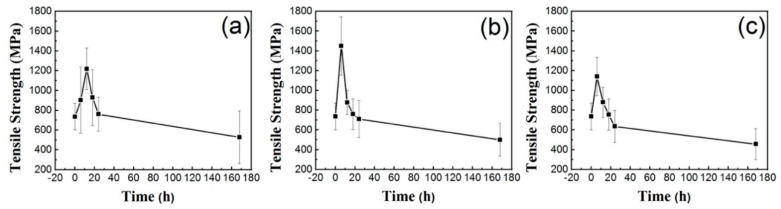
The tensile strength of basalt fiber immersed in seawater solution at different temperatures (**a**) 80 °C; (**b**) 85 °C; (**c**) 90 °C.

The tensile strength of BFs was measured and calculated according to the ISO 11566–1996 standard [23], which is the maximum strength the fiber can withstand before breakage. The tensile strength of basalt fiber after soaking at 80, 85, 90 °C for 0, 6, 12, 18, 24, and 168 h is presented in Figure 2. Results from Figure 2a show that the tensile strength of BFs was continuously increased from 736 MPa to 1219 MPa during the first 12 h after soaking in the seawater solution, and it reduced gradually afterward. A similar phenomenon was detected also in Figure 2b,c, although the maximum tensile strength was reached slightly earlier at 6 h. The tensile strength of BFs will eventually decline to a very low level around 400 MPa after 168 h of soaking at different temperatures.

### 3.3. Scanning Electron Microscopy (SEM) and Energy-Dispersive X-Ray Spectroscopy (EDS)

**Figure 3:**
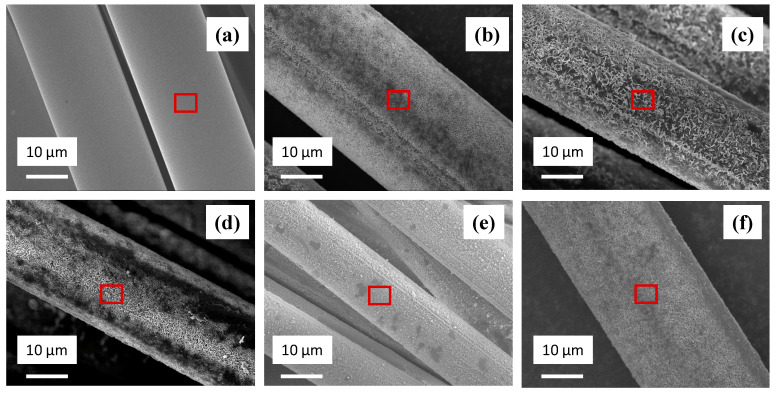
Scanning electron microscopy (SEM) surface morphology of basalt fiber after immersing in seawater solution at 85 °C (**a**) pristine; (**b**) 6 h; (**c**) 12 h; (**d**) 18 h; (**e**) 24 h; (**f**) 168 h.

Figure 3 shows the SEM surface morphology of pristine BFs and basalt fibers after they were immersed in seawater solution for different periods at 85 °C (biggest change in tensile strength). From the visual observation, the surface of pristine BFs in Figure 3a is smooth and with few defects. With the increase of seawater soaking time, the surface corrosion of BFs becomes more serious. The outside surface of BFs starts with the ‘point-like’ corrosion which is shown in Figure 3b, and gradually diffuses to the inside of the fiber and forms a ‘petal-like’ corrosion morphology shown in Figure 3c. Moreover, some fiber materials were peeled off from the BFs which is observed in Figure 2b. Lastly, the surface of BFs in Figure 3f is becoming similar to that in Figure 3b after 168 h of soaking.

**Figure 4:**
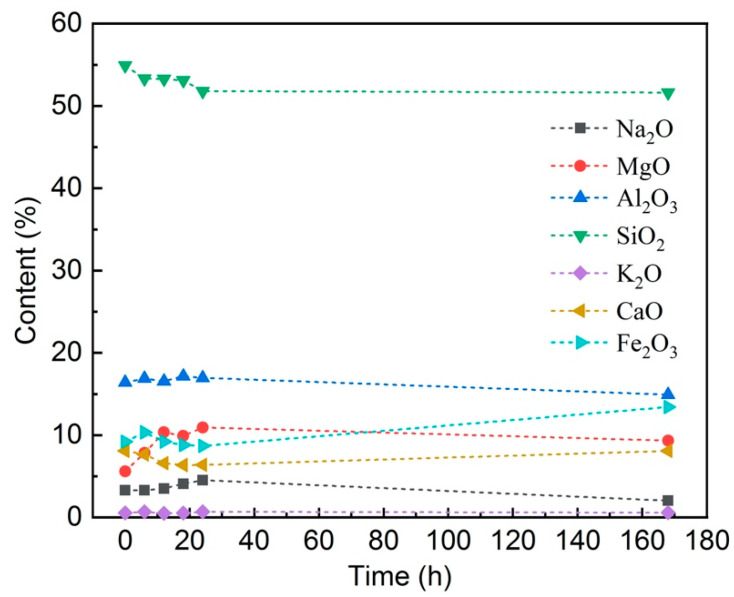
Energy-dispersive X-ray spectroscopy (EDS) analysis of basalt fiber surface after soaking in 85 °C seawater solution for different times (wt%). Dash lines are the guide of eyes.

The EDS analysis of each BF was carried out by selecting a point on the surface (inside the red box), and the results are listed in Figure 4. Results show that most of the elements only have very minor change (<2 wt%) at the first 18 h, whereas MgO owns the biggest change around 4 wt%. With the corrosion proceeding, SiO_2_ decreased around 3 wt% when the soaking time increased to 168 h, which indicates the damage of the former glass network.

### 3.4. Microstructure (Fourier Transform Infrared (FT-IR) Spectroscopy)

**Figure 5:**
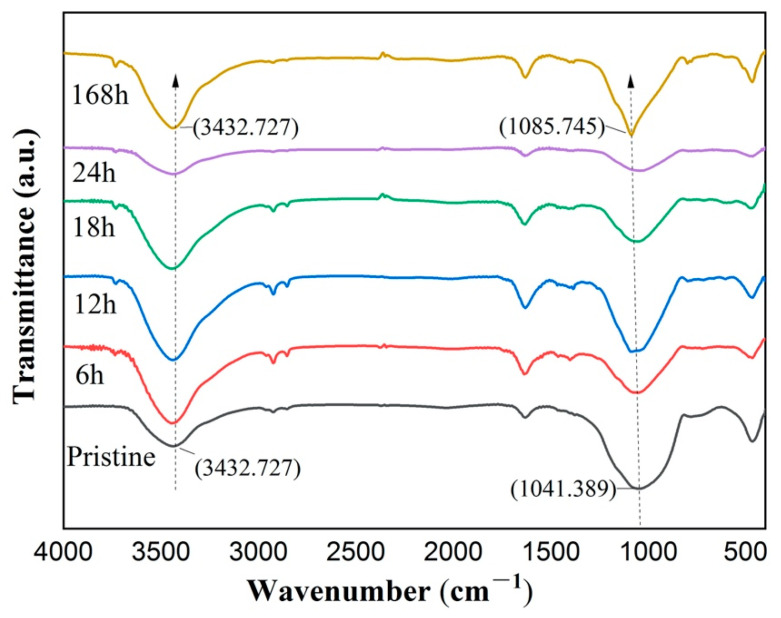
Fourier transform infrared (FT-IR) spectra of basalt fibers after being immersed in seawater solution at 85 °C for different times.

To explore the glass microstructure, the FT-IR spectra of BFs after immersing them in seawater solution at 85 °C for different periods are shown in Figure 5. It can be seen that the peak wavenumber on the right side decreases from 1041 cm^−1^ to 1085 cm^−1^ which is corresponding to pristine BFs and BFs after soaking for 168 h. It is widely accepted that the FT-IR peak near 1050 cm^−1^ represents the antisymmetric stretching vibration of Si-O-Si [24,25]. Thus, this corresponding peak shifts to higher wavenumbers with prolonging of soaking time. On the other hand, the peak at around 3433 cm^−1^ does not change significantly which is associated with the O–H stretching vibration peak in -OH [26].

## 4. Discussion

What happened to the BFs during the first stage (around 18 h) of soaking? The weight-loss ratio decreased and the tensile strength increased after the first stage of soaking. The surface morphology presents a severe surface corrosion structure including cavities and peelings. Meanwhile, MgO has a significant increase after soaking in seawater for around 18 h. We believe all those phenomena are related to each other.

**Figure 6:**
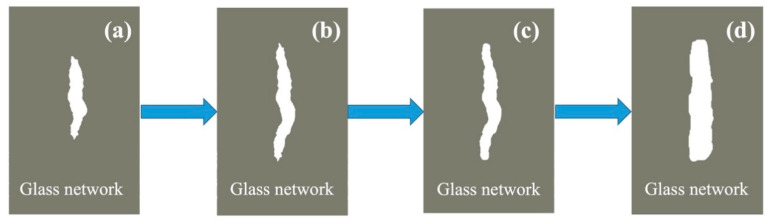
Schematic diagram of the micro-crack blunting mechanism (**a**) pristine; (**b**) microcracks growth; (**c**) tip of microcracks destroyed; (**d**) microcracks blunting.

Micro-crack theory [27] suggests that many tiny cracks or defects are always presented on glass surface. With the proceeding of corrosion (external stress), stress concentration will occur near those cracks and defects, and those cracks will propagate until fracture. Figure 6 describes the mechanism of the micro-crack blunting process [28]. First, microcracks always possess the weakest structure which provides ‘shortcuts’ and promotes the corrosion process (Figure 6a). This will lead to an increase in the weight-loss ratio and the decrease of the tensile strength of BFs. However, the corrosion process provides opportunities for ion exchange between the glass matrix and the seawater solution. One promising piece of evidence is the significant increase in MgO. The ion-exchange process will destroy the tip of microcracks (Figure 6c), which will lead to the blunting of microcracks (Figure 6d). Thus, the micro-crack blunting mechanism is the main reason for the decrease of weight-loss ratio and the increase of tensile strength at the first stage of soaking.

Moreover, the FT-IR results show that, the Si–O–Si antisymmetric stretching vibration peak shifted to the higher wavenumbers with the increase of corrosion time, which represents the depolymerization [29,30] of the Si–O–Si tetrahedral structure. This is direct evidence to support, even the rigid glass network, e.g., Si–O–Si tetrahedral, was also slightly damaged during the corrosion process. The EDS analysis is also in agreement with this conclusion. However, the micro-crack blunting effect is more pronounced than the structure depolymerization at the first stage of soaking (around 18 h), which dominates the tensile strength of BFs.

We note that all the experiments were performed in an accelerated aging method [31,32] which was at a temperature higher than the true seawater environment. Nevertheless, the whole corrosion process at true seawater environment will be the same trend but much slower corrosion rate. Moreover, the mechanism of BFs destruction could be significantly different in different concretes, thus, more chemical and mechanical tests are needed in the future especially for BF composites in seawater environments.

## 5. Conclusions

The effect of seawater environment on chemical durability and tensile strength of basalt continuous fibers was investigated in this work. The main conclusions can be summarized as: (1) the weight-loss ratio of basalt fiber decreases at the first stage (around 18 h) of soaking at 80 °C, 85 °C, and 90 °C and then increases for longer soaking durations; (2) the tensile strength of basalt fiber undergoes the opposite change, which is an increase at the first stage and a decrease later at the same experimental conditions; (3) the enhancement of tensile strength and chemical resistance at the first stage of seawater soaking is due to the ion-exchange induced ‘blunting’ mechanism; (4) the FT-IR and EDS results revealed damage to the Si–O–Si tetrahedral structure during the corrosion process; however, the ‘blunting’ effect dominates the first stage.

## Figures and Tables

**Table 1:** Chemical composition of basalt glass.

Composition	SiO_2_	Al_2_O_3_	Fe_2_O_3_	MgO	CaO	K_2_O	Na_2_O	Others
Content (wt%)	54.90	16.40	9.20	5.60	8.10	0.55	3.30	1.95

**Table 2:** Chemical composition of the seawater solution.

Composition	NaCl	MgCl_2_	Na_2_SO_4_	CaCl_2_	KCl
Content (g/L)	24.53	5.20	4.09	1.16	0.69

## Data Availability

The data presented in this study are available on request from the corresponding author.
